# Prevention and management of malaria during pregnancy: findings from a comparative qualitative study in Ghana, Kenya and Malawi

**DOI:** 10.1186/1475-2875-12-427

**Published:** 2013-11-20

**Authors:** Christopher Pell, Arantza Meñaca, Nana A Afrah, Lucinda Manda-Taylor, Samuel Chatio, Florence Were, Abraham Hodgson, Mary J Hamel, Linda Kalilani, Harry Tagbor, Robert Pool

**Affiliations:** 1 Centre for Social Science and Global Health, University of Amsterdam, Amsterdam, The Netherlands; 2 Centre de Recerca en Salut Internacional de Barcelona (CRESIB, Hospital Clínic-Universitat de Barcelona), Barcelona, Spain; 3 Departamento de Antropología Social, Universidad Complutense de Madrid, Madrid, Spain; 4 Department of Community Health, School of Medical Sciences, Kwame Nkrumah University of Science and Technology, Kumasi, Ghana; 5 College of Medicine, University of Malawi, Blantyre, Malawi; 6 Navrongo Health Research Centre, Navrongo, Ghana; 7 The Kenya Medical Research Institute (KEMRI) and Centers for Disease Control and Prevention (CDC) Research and Public Health Collaboration, Kisumu, Kenya; 8 Research and Development Division, Ghana Health Service, Accra, Ghana; 9 Division of Parasitic Diseases and Malaria, Centers for Disease Control and Prevention (CDC), Atlanta, GA, USA

**Keywords:** Malaria, Pregnancy, IPTp, Insecticide-treated bed nets, ITNs, Malaria case management

## Abstract

**Background:**

In endemic regions of sub-Saharan Africa, malaria during pregnancy (MiP) is a major preventable cause of maternal and infant morbidity and mortality. Current recommended MiP prevention and control includes intermittent preventive treatment (IPTp), distribution of insecticide-treated bed nets (ITNs) and appropriate case management. This article explores the social and cultural context to the uptake of these interventions at four sites across Africa.

**Methods:**

A comparative qualitative study was conducted at four sites in three countries: Ghana, Malawi and Kenya. Individual and group interviews were conducted with pregnant women, their relatives, opinion leaders, other community members and health providers. Observations, which focused on behaviours linked to MiP prevention and treatment, were also undertaken at health facilities and in local communities.

**Results:**

ITNs were generally recognized as important for malaria prevention. However, their availability and use differed across the sites. In Malawi and Kenya, ITNs were sought-after items, but there were complaints about availability. In central Ghana, women saved ITNs until the birth of the child and they were used seasonally in northern Ghana. In Kenya and central Ghana, pregnant women did not associate IPTp with malaria, whereas, in Malawi and northern Ghana, IPTp was linked to malaria, but not always with prevention. Although IPTp adherence was common at all sites, whether delivered with directly observed treatment or not, a few women did not comply with IPTp often citing previous side effects. Although generally viewed as positive, experiences of malaria testing varied across the four sites: treatment was sometimes administered in spite of a negative diagnosis in Ghana (observed) and Malawi (reported). Despite generally following the advice of healthcare staff, particularly in Kenya, personal experience, and the availability and accessibility of medication – including anti-malarials – influenced MiP treatment.

**Conclusion:**

Although ITNs were valued as malaria prevention, health messages could address issues that reduce their use during pregnancy in particular contexts. The impact of previous side effects on adherence to IPTp and anti-malarial treatment regimens during pregnancy also requires attention. Overtreatment of MiP highlights the need to monitor the implementation of MiP case management guidelines.

## Background

In endemic regions of sub-Saharan Africa, malaria during pregnancy (MiP) is a major preventable cause of maternal and infant morbidity and mortality [1]. Malaria during pregnancy compounds or provokes anaemia, which, when severe, increases the risk of maternal death (estimated at around 10,000 deaths annually [2]). Low birth weight (linked to around 100,000 annual infant deaths in Africa [2]), pre-term delivery, congenital infection and reproductive loss are also linked to MiP [3]. Nonetheless, in spite of its associated high burden of morbidity and mortality, MiP was, until recently, recognized as a neglected area of research [4].

Current recommended MiP prevention and control strategies in areas of stable moderate to high malaria transmission include the administration of intermittent preventive treatment (IPTp) with sulphadoxine-pyrimethamine (SP), distribution of insecticide-treated bed nets (ITNs) and appropriate case management [5]. Despite the progress made in the last decade, the coverage of IPTp and ITNs amongst pregnant African women remains inadequate [6]. For proper case management, the most appropriate treatment depends on the malaria species, severity of infection, local patterns of drug resistance, drug availability and gestational age [7]. This, combined with incomplete MiP surveillance data across sub-Saharan Africa [5], complicates estimates of appropriate case management. Nevertheless, given the insufficient availability of diagnostic tests and artemisinin-based combination therapy (ACT) [5] (recommended as first-line treatment for MiP during the second and third trimester), case management is likely to be sub-optimal.

In response to the challenges of MiP prevention and control, a research consortium of 47 partner institutions in 32 countries, was established and is currently conducting a wide range of scientific activities in Africa, Asia, Australasia (Papua New Guinea) and South America [8]. The MiP Consortium takes a multi-disciplinary approach to MiP, bringing together immunologists, epidemiologists, public health experts and social scientists. This paper draws on the results of an anthropological programme of research that forms part of the consortium’s Public Health Impact Group.

The overall goal of the anthropological research carried out under the auspices of the MiP Consortium is to contribute to the development and implementation of appropriate MiP interventions by gaining an in-depth understanding of the social and cultural context of MiP. A review of previously conducted research identified four broad topics that influence the uptake of MiP interventions [9]: concepts of malaria and risk in pregnancy; attitudes towards malaria prevention and treatment; perceptions of antenatal care (ANC) services; and structural factors. These four themes have been widely explored by the MiP Consortium’s Anthropology Team. This article focuses on attitudes towards MiP prevention and management (testing and treatment); two separate articles focus on attitudes towards concepts of malaria and risk during pregnancy [10] and perceptions of ANC [11]. The analysis of the structural factors affecting MiP prevention and control is integral to all three articles.

In recent years, the malaria research and policy community has increasingly emphasized accurate diagnosis to ensure appropriate treatment of malaria. In the absence of diagnostic tests, malaria is commonly over-diagnosed because of its non-specific symptoms: headache, fatigue, abdominal pain, muscle and/or joint aches, fever, chills, perspiration, vomiting and malaise [12]. Therefore, as a result of concerns about overuse of malaria drugs, the 2010 WHO Guidelines for the treatment of malaria recommended diagnosis by microscopy or rapid diagnostic test (RDT) for all persons suffering from suspected malaria prior to treatment [12]. Moreover, there is overlap between malaria and women’s other health complaints during pregnancy. Thus diagnostic confirmation of MiP is particularly important for appropriate management [13] and is a key issue for this article.

To provide insight into the social and cultural context to the uptake of interventions for malaria prevention and control, this article addresses the following research questions: what are the attitudes and behaviours towards MiP prevention and management amongst pregnant women, healthcare staff and other community members in four sites in Ghana (northern and central), Kenya (Nyanza Province) and Malawi (southern region); how does the social and cultural context influence these attitudes and behaviours; and what are the implications of the attitudes and behaviours for the design of effective MiP interventions? To identify and examine relevant issues that might otherwise be taken for granted, a comparative approach is taken and data are presented from four sites.

## Methods

The results presented in this article are drawn from a comparative study conducted at four sites in three countries. The study was undertaken by a team of researchers whose members were based across the sites and in Barcelona (Spain).

### Settings

The study incorporated one country from each of the three main regions of Sub-Saharan Africa: Ghana in West Africa, Kenya in East Africa and Malawi in Southern Africa. Two sites with important regional specificities were selected in Ghana for several reasons: to collect data in at least one site of each of the MiP Consortium’s main treatment and prevention activities; to include areas with different patterns of malaria transmission; and to examine intra as well as inter-country variation.

In central Ghana, fieldwork was conducted in two districts of the Ashanti Region: Ejisu Juaben and Ahafo Ano South. In both districts, agriculture is the main productive activity and there is a significant proportion of internal migrants, in addition to the majority ethnic group, the Asante [14]. At this site, malaria transmission is moderately high and occurs throughout the year with peaks during the rains in May-October [15]. In each district, data collection was conducted at the district hospitals, two to three health centres and several smaller clinics.

In northern Ghana, Upper East Region, the fieldwork sites were located in Kassena-Nankana District. This area is part of the Sahel and experiences only one annual rainy season during which people grow millet, maize and vegetables for subsistence. During the rest of the year, part of the population migrates to other regions. The Kassena and the Nankani, make up almost 90% of the population of the district [16]. Here, malaria transmission is perennial but there is a seasonal pattern with a transmission peak that coincides with the major rains (May to October) and the low rates of infection during the dry season [17]. Data were collected at a district hospital in Navrongo, (the capital), and outreach community-based services, which are common throughout the area.

Fieldwork also took place in Chikwawa and Blantyre Districts, in the southern region of Malawi. The main ethnic groups in Blantyre District are Chewa and Yao, whereas in Chikwawa they are Manganja and Sena. Most of the women in the area cultivate crops for subsistence and sale at the market. Both districts are in areas of high perennial malaria transmission [18]. Fieldwork took place at three hospitals, and six healthcare centres that provide ANC services to the women in these areas.

Finally, in Kenya, fieldwork was carried out in Siaya District (Nyanza Province) where the principal ethnic group, the Luo, make up over 95% of the population. Livelihood activities include subsistence farming of maize, sorghum, millet and cassava. As a result of the relatively limited employment opportunities, migration to urban centres is common, particularly to Kisumu, the nearest city. Malaria transmission is high and perennial [19] with the greatest disease burden borne by children and pregnant women. Data were collected at the district hospital and smaller health facilities where ANC is delivered.

At each of the sites, various clinical and non-clinical studies of MiP prevention and control interventions have been undertaken. Some of these studies overlapped with data collection for this research. Therefore, during data collection and analysis, efforts were made to exclude experiences of MiP prevention and control within clinical or non-clinical research. Furthermore, throughout this article, for reasons of brevity, the sites are referred to as “Kenya”, “Malawi”, “central Ghana” and “northern Ghana”. This shorthand should not however be interpreted as any attempt at regional or national generalization.

### Data collection

An anthropological approach was taken to data collection. This entailed year-long (or longer) periods of fieldwork at each site, a range of data collection activities, including narrative and observational techniques (see Table 1 for a full list of data collection activities), and a flexible, reflexive and iterative process of tool design, data collection, and analysis. The use of multiple data collection tools with heterogeneous respondents ensured that findings could be triangulated and their reliability tested. To reduce the possible influence of individual bias on the study findings, at each site, several researchers collected data.

**Table 1:** Respondents

**Data collection activities**	**Respondent type**	**Central Ghana**	**Northern Ghana**	**Kenya**	**Malawi**	**Total**
Free listing and sorting	Community members	12	16	17	24	**59**
	Pregnant women	10	10	7	11	**38**
	Health providers*	10	11	6	5	**32**
In-depth interviews	Pregnant women	84	64	69	68	**285**
	Health providers*	33	34	17	21	**105**
	Relatives	26	29	20	16	**91**
	Opinion leaders	12	12	10	12	**46**
Case studies	Pregnant women	19	18	12	18	**67**
Focus group discussions	Community members	10	16	9	16	**51**

*Includes healthcare staff involved with the provision of ANC at health facilities and TBAs working in the communities.

Fieldwork was carried out between April 2009 and August 2011, and lasted from one year in Malawi to more than two years in central Ghana. Assisted by two Barcelona-based researchers (AM and CP), fieldworkers spent extended periods of time in the settlements where data were collected and recorded their experiences of participant observation in field diaries. In the first phase, at each site, using free-listing and sorting exercises, the research team explored the main problems that pregnant women experience. Later, interviews and group discussions were conducted, several women (case studies) were followed and interviewed several times over the course of their pregnancies, and observations were carried out in the communities and at local health facilities. The language used to interact with informants depended on their preferences (English and different local languages). In-depth interviews and group discussions were recorded, transcribed and translated into English.

In-depth interviews tended to start with broad research questions related to pregnancy and ended with questions about malaria in pregnancy and experiences with malaria prevention and control. In contrast, group discussions often started with general questions about malaria, focusing later on groups particularly vulnerable to malaria and finalizing with malaria prevention and control. Other themes, related to MiP, such as miscarriage, stillbirths, pre-term deliveries, birth weight and anaemia – and their causes – were also explored during fieldwork. Data collection and analysis were carried out in parallel allowing the incorporation of emerging themes in the design of the tools, and questions’ redefinition and attuning.

Members of the Barcelona-based research team made quarterly visits to the study sites. During these visits, a process of debriefing and reflection took place with fieldworkers. The Barcelona-based researchers were also able to participate in data collection, and provide ongoing training.

### Respondents

Five main categories of respondents were interviewed (Table 1): pregnant women, their relatives, community members, opinion leaders and healthcare providers. Purposive sampling was used to ensure the interaction with a wide range of experiences. Married and unmarried pregnant women of a range of ages, parities and gestational ages from across the different settlements (within the field sites) were interviewed. Relatives included mainly mothers, mothers-in-law and husbands of the pregnant women. The sample of opinion leaders was made up of a variety of religious leaders, traditional and political authorities, and relevant women in the local communities. Finally, ANC staff, pharmacists and drug sellers, traditional birth attendants (TBAs), and other healers (who attended to pregnant women or dealt with malaria) were interviewed at each site. Respondents were identified in ANC clinics and via contacts in the local communities, which developed as fieldwork went on. The final number of participants was a result of the directed sampling and the point of saturation, whereby no further novel insights were identified from interviews.

### Data analysis

At each site, a first phase of data analysis ran in parallel to data collection. Using Atlas.ti 6, flexible codebooks were developed and revised using a combination of established categories based on the original research questions and themes that emerged from the data. Particular attention was paid when analyzing the interviews with case studies to identify changes in a woman’s responses over the course of her pregnancy, for example, with regard to ITN use. The preliminary results obtained from this site-specific analysis were compared and discussed amongst the members of the team in periodic meetings throughout data collection. In a second phase, data associated to the codes relevant to malaria in pregnancy perceptions, were extracted, collated and discussed between authors one and two, looking at the similarities, differences and variations between and within the different sites.

### Ethics statement

Overall ethics clearance was obtained from the Clinical Research Ethics Committee, Hospital Clinic-University of Barcelona. Separate local ethics clearance was obtained at each site: in Ghana, clearance was obtained from the Institutional Review Board of the Navrongo Health Research Centre, Navrongo and the Committee on Human Research Ethics, Kwame Nkrumah University of Science & Technology, Kumasi; in Kenya, clearance was obtained from the Institution Review Board of Centers for Disease Control and Prevention, Atlanta and from the National Ethics Review Committee, Kenya Medical Research Institute, Nairobi; and in Malawi, clearance was obtained from the College of Medicine Research and Ethics Committee. As approved by all ethics review committees and institutional review boards, informed consent was obtained orally from study participants. Oral rather than written informed consent was obtained because the study procedures posed minimal risk to study participants and to avoid the possible negative influence of a written consent on rapport between researchers and respondents. With the agreement of participants, verbal consent was voice recorded prior to each interview or focus group discussion.

## Results

### Prevention

#### ITNs

Sleeping under an ITN was generally recognized as the main way to prevent malaria (or the local illnesses that overlapped with biomedically defined malaria at each site, which, although not addressed specifically here, are discussed in a separate article [10]). The availability and use of ITNs however differed across the four sites (see Table 2).

**Table 2:** Insecticide-treated bed nets (ITNs) for pregnant women: policies, availability and preferences

**Site**	**Policy**	**Shortages**	**Availability and use**
**Kenya**	Free delivery at health facilities	No	Pregnant women report sleeping under ITNs
			Pregnant women claim ITNs are not provided
			Observations in houses confirm presence of ITNs
**Malawi**	Free delivery at health facilities	Observed	Pregnant women report sleeping under ITNs
**Ghana (northern)**	Sold in health facilities at subsidized prices	Observed	Cases of seasonal use of ITNs
			Pregnant women complain about costs of ITNs
**Ghana (central)**	Sold in health facilities at subsidized prices	No	Pregnant women report saving for use after delivery

#### Availability and access of ITNs

In Ghana, ITNs were sold in the health centres at ANC at a subsidized price but purchases were rarely observed. Moreover, in Northern Ghana, shortages of nets were encountered. No national mass ITNs distribution campaigns were observed during fieldwork, however, a non-governmental organization distributed free ITNs to the local population and a limited number of our informants in northern Ghana received an ITN.

In Malawi, insecticide-treated bed nets were distributed free of charge to pregnant women at health facilities on two occasions: at first ANC visit, and once after delivery. Observed shortages however supported pregnant women’s claims of not always receiving an ITN. Bed nets were sought-after items in Kenya and, although healthcare staff reported providing ITNs to all women who attend ANC (and observations suggested that they were plentiful at health facilities), there were complaints from pregnant women of ITNs not being handed out as part of ANC.

#### ITN ownership and use

At all sites, pregnant women reported a demand for ITNs. Although they complained about their price, Ghanaian women reported sleeping under ITNs. However, in central Ghana there were women who, in spite of owning an ITN, only used it after giving birth; these women considered their newborns to be at greater risk of malaria and also valued new possessions to mark the birth of a child. In northern Ghana, women used ITNs in the wet season because mosquitoes were present and they slept indoors. However, in the dry season, due to the heat, outside sleeping was the norm and ITNs were therefore not used. In Kenya and Malawi, although household sleeping arrangements and the lack of space in dwellings prevented some pregnant women from sleeping under ITNs (particularly if children were given priority), respondents generally reported that they used their ITNs, sometimes sleeping alongside their youngest children. Moreover, in Kenya, ITNs were often directly observed hanging in respondents’ dwellings. The value that Kenyan respondents placed on ITNs was also linked to their observed multiple uses: for example, as garden netting, fishing nets, latrine doors, crop protectors and decorative wall hangings.

During fieldwork, there were also very scattered reports of opinions that might deter women from sleeping under ITNs. In Kenya, there were infrequent references to feeling hot and suffocated when under the ITN, and similar comments about heat discouraging people from sleeping under ITNs in Malawi and Ghana. In Malawi and Ghana, although rare, criticisms were made of the insecticides used to treat the ITNs.

### IPTp

#### Availability of IPTp

Policies regarding IPTp delivery varied across the sites: in Malawi, women were to be administered two doses of IPTp, whereas, in Ghana, policy stipulated a three-dose regimen. In Kenya, at least two doses were to be delivered at monthly intervals after quickening. At all sites, shortages of SP for IPTp were not encountered and healthcare staff described them as exceptional occurrences.

#### Knowledge of IPTp

In central Ghana and Kenya, pregnant women did not usually associate IPTp with malaria, whereas, in Malawi and northern Ghana, IPTp was associated with malaria, but not always with prevention. In central Ghana, only a minority of pregnant women recalled being given pills to prevent malaria; when probed about the tablets that they received during ANC visits, the majority did not know what they were for or interpreted them as intended for general pregnancy care. Moreover, younger mothers were less aware of the drugs provided.

Interviewer (I): *Why do you think they gave you those three medicines?*

Respondent (R): *I didn’t ask them. When they gave them to me, I took them over there. So I didn’t ask them why they gave them to me but I know they gave them to me because of the pregnancy.*

(Central Ghana, in-depth interview with a pregnant woman, 24 years old, one child)

Kenyan pregnant women were often unfamiliar with IPTp as malaria treatment or prevention. Although a small minority of respondents recalled taking “Fansidar” (the brand name for SP) during ANC visits to prevent malaria, those who did not, when probed about the white tablets supplied during ANC, reported that they were de-worming tablets, tablets to cure their (mild) malaria/fever, multi-vitamins or they simply did not recall receiving them (in spite of healthcare staff reporting that all women receive IPTp). Although a lack of communication between healthcare staff and pregnant women during ANC was observed at this site (and at other sites), the Kenyan women who said that they asked health care staff specifically about the white tablets reported that they received information.

In northern Ghana, there was greater awareness of malaria drugs being administered during ANC: around half of the interviewed pregnant women referred to the malaria drugs, but most were unaware that they were intended as prevention. Pregnant women’s knowledge of IPTp was also high in Malawi: most respondents were familiar with “Fansidar” as an anti-malarial drug given to pregnant women and, when administered during ANC, it was considered to provide protection from malaria for the mother and the unborn child and/or treatment for the pregnant woman’s malaria.

I: *What about the Fansidar they gave you, what was it for?*

R: *It protects against malaria but also deals with general body pains.*

(Malawi, in-depth interview with a pregnant woman, 25 years old, two children)

#### Attitudes towards IPTp

The lack of awareness of IPTp in Kenya and central Ghana complicated attempts at assessing attitudes to IPTp or SP. However, regardless of whether pregnant women were familiar with IPTp, complaints of side effects linked to the medication received during ANC visits (largely nausea and dizziness) were uncommon at these sites. In Malawi and Northern Ghana, direct complaints about side effects of IPTp, especially vomiting, were more common. This was in spite of women’s greater efforts to reduce the possibility of side effects at these sites: here, women were often advised by healthcare staff to eat a meal before receiving IPTp to prevent nausea or vomiting. These complaints did not however necessarily lead to non-compliance because IPTp was generally accepted (albeit sometimes begrudgingly) as part of the package of ANC interventions, which was collectively viewed in a positive light.

I: *Do you think that those medicines have disturbed some pregnant women?*

R: *Yes, some women swallow and go out and vomit it away but I have never swallowed it and vomited it out.*

(Northern Ghana, in-depth interview with a pregnant woman, 22 years old, one child)

#### IPTp-related behaviours

Observations highlighted how IPTp was not universally administered according to the directly observed treatment (DOT) protocol. This was often a result of drinking-water being only available outside of the consultation room: freely available from drinking fountains in communal spaces or purchased from vendors on or close to the health facility premises. However, at one health facility in central Ghana, DOT was contingent on other factors: prior to administering IPTp, healthcare staff first enquired whether pregnant women had eaten. If they had eaten prior to attending the health facility, the SP was administered during the consultation under DOT. However, if they had not eaten, the women were instructed to take the SP away with them and to take it after eating. These staff, therefore, prioritized the Ghanaian policy guideline that stipulates women should have eaten prior to receiving SP (which is contradicted by more recent WHO guidelines [20]) over that which specifies DOT for IPTp [21].

Instances of IPTp non-adherence were however encountered. In Kenya, for example, DOT was not always followed because the drinking water fountain was located in the waiting area and a woman was observed slipping her tablets into her handbag on leaving the consultation room). When asked about this, she explained her non-adherence in terms of previous experiences of vomiting after taking the same tablets. She was also unaware of the purpose of the medication. At the other sites, there were also indirect reports (and one direct) of non-adherence, and the following quotation hints at non-adherence occurring even in cases of DOT.

I: *Were you given some drugs to take instantly at the initial visit?*

R: *Yes*

I: *What was the colour?*

R: *White*

[…]

I: *Were you told what it was meant for?*

R: *No*

[…]

I: Did you have any problems when taking them?

R: I always vomited

[…]

I: Did you tell [the healthcare staff] that the medicine was making you vomit?

R: No

I. So did you take them and vomit again?

R: I didn’t take them again even though I was given them.

I: But you are always asked to take them straight away?

R: I was given the drugs and water to take but I kept [the tablets] in my purse.

I & R: [Laughter].

(Northern Ghana, in-depth interview with a pregnant women between 20 and 25 years old, one child)

Generally though, according to healthcare staff and pregnant women, even without DOT and even though they may have been unaware about the name or purpose of the medication, women took the SP when it was given to them. This was even the case in both sites in Ghana, where women had to buy water to swallow the tablets from vendors in health facilities for a small fee (US$0.05) that women viewed as an insignificant part of the total cost of ANC (including transport costs etc.). Indeed, during ANC visits, women followed instructions from healthcare staff that provided the interventions (see [11] for further details regarding ANC attendance).

### Case management

#### Malaria diagnostic tests

Although generally positive, attitudes towards and awareness of malaria testing varied across the sites. Pregnant women, and the community in general in central Ghana, accepted healthcare staff as providing accurate diagnoses of their health complaints, whether blood analyses were carried out or not. Women could however not always recall the purpose of the tests – for malaria or otherwise – during clinic visits.

I: *How would you know a pregnant woman has malaria?*

R: *It is only when she goes to the hospital would they be able to tell whether she has malaria or not because pregnant women are often weak. So you might even think she is sick when she is not.*

(Central Ghana, in-depth interview with the husband of a pregnant woman, 35 years old)

In northern Ghana, pregnant women identified contradictions between the messages provided in health facilities and their own experiences of malaria: some women complained of being frequently told that malaria was the reason they were feeling unwell, whereas, on other occasions, they thought that they had malaria but were not given treatment. As the quotation below suggests, ambiguity was often linked to the breadth of the local illness concept that approximated to biomedically defined malaria (as is discussed in more detail elsewhere [10]).

I: *Did you not have malaria at a point in time?*

R: *Ok. There was a time when I went and told [the healthcare staff] that I had malaria and they told me that we should not always say that we have malaria, but we should just say that we are sick, because we cannot tell whether it is malaria or not. But I know that I had malaria because I was vomiting.*

I: *Did they give you any malaria drugs?*

R: *Yes they gave me some. They made me go and do some test before they gave me that medicine*.

(Northern Ghana, an in-depth interview with a pregnant woman, 29 years old, one child)

At both sites in Ghana, health professionals described the importance of diagnostic tests for accurate malaria diagnosis. In these settings, however, health professionals also explained that infants and pregnant women were exceptions to the new policy on malaria case management that stipulated a positive malaria test prior to administering anti-malarials. Hence, pregnant women were observed receiving anti-malarials without being tested (even if a test was available) or despite a negative malaria test result.

In Malawi, pregnant women viewed malaria diagnostic tests as ambiguous. Although not always available, when they were available, positive results were often trusted and seen as confirmation of a diagnosis. In cases of a negative result, the test was sometimes viewed as missing the infection. The subsequent lack of treatment also disappointed women who had assumed that they were suffering from malaria and expected to be treated. However, the following quotation from a focus group is one example of the reports of treatment in spite of a negative test result.

I: *Can one have malungo (malaria) without knowing?*

All: *Quite.*

[…]

R5: *We get tested and then you know that you have malungo even when you go [to the clinic] for another disease and not malungo.*

I: *Is it possible to feel that you have malungo and at the hospital they do not find it?*

R1: *Yes, it’s possible*.

All: *Yes.*

I: *So what happens when you have all the symptoms and they do not find the malungo?*

R5: *They give AL (artemether-lumefantrine).*

All: *Yes.*

(Malawi, a group discussion with local women)

In contrast, interviewed Malawian healthcare staff only referred to treating after a positive result. Moreover, malaria-like symptoms were said to be common amongst pregnant women, but the rapid diagnostic test confirmed a minority of these cases as malaria (one health worker referred to one in ten).

Kenyan respondents generally valued diagnostic tests for malaria (and other diseases): they viewed them as accurate and associated them with more effective treatment. Indeed, one pregnant woman’s husband stressed the need to travel the extra distance to the district hospital to ensure access to diagnostic tests. Nonetheless, respondents described having “malaria”, particularly mild “malaria”, without having obtained a diagnosis (either from a health worker based on clinical presentation or the result of a blood test). This was linked to the broad illness category of “malaria” that respondents described, which did not necessarily match the biomedical definition [10].

For the most part, malaria tests were only available free of charge through the laboratories of medical research institutes that were carrying out clinical studies within the grounds of health facilities. In smaller health centres, a malaria test was only available with the assistance of staff from the medical research institutes, who tested non-study patients when requested by healthcare staff or when severe malaria was suspected. At the district hospital, although malaria tests were sometimes available in the Ministry of Health laboratory, charges were commonly levied for these tests. There were therefore reports of healthcare staff administering anti-malarials without a test.

Sometimes you may go to these [drug] shops and tell them your problems for example, I have a headache but they will not do a lab test so they will just give you medicine thinking that it is malaria but it is not. Now it is good to go to the hospital and get tested so that you may know the problems that you are suffering from

(Kenya, in-depth interview with a pregnant woman, 20 years old, two children)

Indeed, Kenyan healthcare staff viewed malaria – symptomatic and asymptomatic – as common amongst pregnant women. In light of this, a diagnostic test was viewed as essential to confirm the diagnosis.

R: *At least, out of all the admissions, you will find that eight out of ten will have malaria. Almost all. And half of them never feel ill. So when you take the temperature that is when you realise but they don’t complain. You see the [blood] slide.*

I: *So you are saying that eight out of ten have malaria and most of them are not complaining.*

R: *Yeah most of them don’t complain, they just see it as part of the discomfort of pregnancy but when now you are doing your research, and doing the blood tests, they will say “ah, I don’t even have malaria”, and you say let’s check. And you check and you will see and have to treat, so that’s malaria.*

(Kenya, in-depth interview with a healthcare provider)

### Treatment

#### Type of anti-malarials

Across the four sites, all respondent types viewed biomedicine as the primary treatment option for malaria, particularly severe malaria. Although some herbal remedies were described, respondents reported their use to be infrequent. In Malawi, references to non-biomedical treatments for malaria were very scarce. In Kenya, poverty was said to be a reason for pregnant women using herbal remedies as a last resort for malaria. In central Ghana, herbal malaria remedies were used, particularly in rural areas by uninsured adults (to avoid the costs of both transport and medical care) and on other occasions for cases of mild malaria in addition to biomedical treatments. At this site, the use of non-biomedical remedies during pregnancy was common to ensure a safe delivery and a healthy baby. However, no remedy specifically for MiP was reported. In northern Ghana, adults and children were regularly given herbal malaria treatments, but pregnant women did not use them because of their bitter taste, which was viewed as a cause of miscarriage.

I: *Is there herbal medicine for paa (malaria)?*

R: *Yes it is there, but in this community they hardly give herbs to pregnant women, because they are afraid, they don’t know what is inside the stomach.*

I: *If you are not pregnant and you have paa, can you use the local medicine?*

R: *Yes, you can use the “soli”, a long tree, and boiled neem leaves. You use it to cover yourself, drink and bathe.*

(Northern Ghana, In-depth interview with a pregnant woman, more than 35 years old, 4 children)

#### Availability of and access to anti-malarials

Anti-malarials, including SP, were available in drugstores at both sites in Ghana. However, during fieldwork there were no reports or observations of pregnant women buying them; in theory, health care is free to pregnant women (though they sometimes are faced with charges [11]) and they therefore preferred to seek care at health facilities. In Malawi, ACT was not easily available, except in health facilities. Moreover, although at the beginning of fieldwork, SP was available in grocery shops, it was later prohibited and therefore increasingly unavailable as stocks ran out. In both countries, women acknowledged buying and taking painkillers, mainly paracetamol, without prescription for mild symptoms of pregnancy and/or malaria. In Kenya, the majority of pregnant women and other respondents maintained that pregnant women should to go to clinic to be prescribed drugs for (especially severe) malaria. However, women admitted having bought drugs from local pharmacies without a prescription or taking other medication that was not prescribed for them (due to the “mildness” of the malaria, the high price of the drugs recommended by healthcare staff, or a reluctance to visit a health facilities, because of cost or other reasons).

#### Knowledge and understandings of anti-malarials

In Ghana, pregnant women did not recall the names of anti-malarials, but some could describe the colours of the tablets, the doses and/or the packaging. In Malawi, the names of anti-malarials were used even if respondents identified them incorrectly. Malawian respondents also viewed the effectiveness of a particular anti-malarial as dependent on the individual: certain tablets were said to suit some individuals better than others. And there was a reasonable consensus amongst respondents regarding which “malaria medicines” (even if not anti-malarials) were appropriate for pregnant women: “Fansidar” (SP) was mainly considered the anti-malarial for pregnant women and a small proportion of pregnant women stated that artemether-lumefantrine (AL), quinine, bactrim, penicillin and ibuprofen were too strong to be used during pregnancy and that they can harm the unborn child. Other women accepted AL as treatment for pregnant women, provided that it “suits” the individual.

There were Kenyan respondents who recounted a list of drugs to treat “malaria” and others who only mentioned one or two. Their lists included painkillers or cold remedies, often intended for mild illness episodes that were labelled using the local illness that overlapped with biomedically defined malaria [10]. Indeed, across all the sites, women reported using paracetamol (often termed “panadol” or “para”) and aspirin for mild symptoms of pregnancy and/or malaria. There were even reports of Ibuprofen use during pregnancy. Kenyan women also mentioned Coartem (AL) and quinine, which tended to be associated with severe malaria. Respondents had varied opinions about taking “malaria drugs” during pregnancy: there were reports of a need to consult healthcare staff, yet there were also cases of pregnant women self-medicating with anti-malarials. One woman was unsure about taking bitter drugs during pregnancy and another was wary about taking “many drugs”.

Although women generally reported following the advice of healthcare staff, in Kenya, the availability and accessibility of medication – including anti-malarials – could influence where women received treatment. For example, one Kenyan woman attended a health facility for an acute illness and sought assistance where ANC is normally provided. However, ANC was not offered at that time, and the healthcare staff who examined her instructed her to pay for the anti-malarials. Unable to pay, she visited a drug store where she bought drugs more cheaply. Such examples were however not observed nor reported in Malawi or Ghana. Furthermore, in general, although compliance with anti-malarial treatment regimens was not observed directly, Kenyan women’s reports of anti-malarial use varied: there were at least two reports of women using ACT that they already had at home, for example, that had been previously prescribed to a young child. Some women however emphasized the need to follow the instructions from healthcare staff because of the dangers of taking medication during pregnancy.

In Ghana, the side effects of anti-malarials and the advice of health staff influenced whether pregnant women completed the prescribed treatment course. In total, four women were identified who did not complete their prescribed treatment courses. For one of these women, a health worker’s advice about sweetening medication caused confusion: the woman associated the sweet taste with malaria and was therefore deterred from completing the treatment course. In both Ghana and Malawi, health talks and the advice given individually to pregnant women at ANC included warnings about self-medication and healthcare staff expressed concerns about the use of non-prescribed drugs and traditional remedies. However, in Ghana, health messages did not focus on adherence to prescribed anti-malarial regimens.

## Discussion

Across all the sites, respondents recognized that sleeping under an ITN was a way of preventing malaria. Even though respondents at all sites offered additional explanations (such as poor hygiene) for a bout of malaria (or the local illness that overlapped with biomedically defined malaria (see [10] for further details), mosquitoes were reported to be the main cause. The connections that respondents made between ITNs use and malaria prevention were therefore unsurprising. Malaria was also viewed as a common disease for pregnant women, and considered to be a cause (along with other contributing factors) of miscarriage [10].

In addition, negative attitudes towards ITNs were rare and there were no specific objections to their use during pregnancy. This finding, in varied social and cultural contexts with different mechanisms of ITN distribution, contrasts with several previous studies that have highlighted health concerns linked to ITN use during pregnancy, particularly with regard to the impact of the insecticide treatments on the unborn child [22-25]. These studies were carried out prior to the distribution of long-lasting ITNs and it was often the insecticide, and process of re-treatment, that provoked such concerns. One multi-site study in Kenya highlighted additional negative attitudes towards ITNs including complaints about distribution campaigns targeting pregnant women and infants [26]. Such comments were attributed to a lack of information linking increased malaria vulnerability with ITN distribution [26]. By contrast, respondents in this study often highlighted pregnant women’s increased risk malaria [10] and no such negative comments about the targeting of pregnant women were encountered.

The broader meanings associated with ITNs had varying implications for their use. In central Ghana, women viewed ITNs as a consumer good and therefore left them unused during pregnancy, hanging them up for the first time to mark the birth of the child. Kenyan respondents also valued ITNs as a household item with multiple uses, such as protecting crops from birds. However, in this context, the multiple uses of ITNs did not necessarily prevent pregnant women from sleeping under them. These findings are a reminder of how health technologies (in this case, intended by its designers for malaria prevention) can take on quite different meanings and usages. This is particularly the case in resource-poor contexts where material goods are scarce and this scarcity can foster innovation.

More practical issues also had implications for ITN use during pregnancy. In Kenya, occasionally, sleeping arrangements together with the prioritization of children sleeping under ITNs prevented pregnant women from using the ITN that they received at ANC. In northern Ghana, ITN use was seasonal, depending on the presence of mosquitoes and temperatures low enough to sleep indoors. In Malawi, shortages of ITNs at health facilities limited the number of women who received free ITNs and their use. However, when available, Malawian women reported sleeping under ITNs.

As previous research has also highlighted [9,15,27], knowledge of IPTp varies across different contexts. In central Ghana and Kenya, pregnant women did not usually associate IPTp with malaria, whereas, in Malawi (as has also been identified in previous research [28]) and northern Ghana, it was more often linked to malaria, but not always prevention. Reports of side effects linked specifically to IPTp were therefore more prominent at these sites and, in northern Ghana, vomiting was particularly associated with IPTp, and, therefore, seen as one negative aspect of ANC. However, vomiting did not lead directly to future non-compliance with IPTp or discourage ANC attendance: often, regardless of side effects and without supervision women took their malaria prevention, “Fansidar” or “white tablets” because it was a component of the ANC package, and in an effort to follow the instructions of healthcare staff – see [11] for more detail of ANC at the same sites. Similar confidence in healthcare staff’s instructions about IPTp has also been identified in The Gambia [27] and Uganda [29]. Even so, the women who did not adhere to IPTp – and there were suggestions that this occurred even if administered under DOT – did so largely because of previous negative experiences. The instances of IPTp non-compliance underscore a need for further in-depth (observational) research on compliance in real-world settings to ensure that uptake of IPTp is not overestimated.

Optimal treatment of MiP was hindered by a lack of available malaria diagnostic tests and by negative test results being ignored. Pregnant women, along with other community members, generally viewed healthcare staff as the authoritative source of malaria diagnosis, particularly severe malaria. Moreover, malaria tests were generally valued as a way of confirming the diagnosis. However, even if available, the tests did not entirely dispel uncertainty around the presence or absence of MiP. The non-specific nature of its symptoms has prompted recent policy recommendations to test for malaria prior to administering treatment [12]. Pregnancy however further complicates the clinical diagnosis of malaria because there is overlap between the symptoms of non-severe malaria and what women often consider to be normal pregnancy experiences [10] or related illness [13]. Although a positive test result resolved any uncertainty for healthcare staff and pregnant clients, a negative result was not so conclusive: in Ghana and Malawi, observations and women’s reports suggested that there instances of treatment in spite of a negative result.

Ghanaian healthcare staff asserted that pregnant women were exceptions to the policy of testing prior to treatment and provided treatment based on symptoms even when a malaria test result was negative. This is however a misinterpretation of the relevant policy document, which states that, in the absence of laboratory diagnosis, pregnant women with clinical symptoms of malaria should not be denied anti-malarials because the risk of not treating far outweighs the risks associated with overtreatment [21]. A lack of confidence in malaria tests and reliance on symptoms is however well-documented in other African contexts [30,31]: for example, in northern Tanzania, overtreatment of malaria in general was linked to a range of factors, including healthcare staff members’ assumptions about malaria being the most important disease and patients’ expectations [30]. Overtreatment of MiP in Ghana was linked to a (mis)interpretation of national policy, yet the findings offer little insight into how this came about. However, the emphasis placed on MiP and its deleterious impact on the health of mother and child could have contributed to the better-safe-than-sorry approach. The reports of treatment in Malawi were however, more indirect. Healthcare staff only referred to treatment after a positive result, even if such tests were not readily available. Pregnant women who suspected malaria but received a negative test result were disappointed when they did not receive anti-malarials, but the data offer no insight into whether this led healthcare staff to provide treatment.

With regard to malaria treatment, pregnant women generally reported following the advice of healthcare staff. However, although scattered, there were instances of women ignoring such instructions or self-treating for malaria without seeking diagnosis at a health facility. In Ghana, where there were four cases of women who did not complete their treatment course, side effects played a role, as did confusion about the advice from healthcare staff, particularly if it contradicted ideas about malaria causation. In spite of such cases, health messages tended to focus on the use of non-prescribed and non-biomedical treatment during pregnancy and little emphasis was placed on adherence to prescribed anti-malarial regimens. The instances encountered in Kenya of women self-treating with drugs from other sources stood out from the other sites. Yet, although similar findings have been made at another Kenyan site [32], it is unclear to what extent they were indicative of a more systematic trend of self-medication. At all the sites, women made use of paracetamol and other cold remedies to combat the mild symptoms associated with pregnancy and/or malaria. Such vague ideas of “malaria” medication (presumably linked to the breadth of the local illness that overlaps with malaria [10]), can contribute to inappropriate use of anti-malarials and other “malaria drugs” through self-treatment or non-adherence to prescribed treatment regimens.

A preference for anti-malarials that “suit” an individual was more prominent in Malawi than at the other sites. Such ideas about compatibility were perhaps the most extreme examples regarding the role that personal experience plays in attitudes and behaviours towards MiP interventions. Because pregnancy is generally considered to be a particularly vulnerable bodily state and one in which women experience a range of health complaints [10], it is unsurprising that women display a preference for health interventions that are viewed as not contributing further to the negative symptoms of pregnancy. Indeed, these experiences can – albeit rarely – override women’s typical adherence to the instructions that healthcare staff provide.

### Strengths and limitations

The strengths of this study are intertwined with the anthropological approach: fieldwork over a one to two year period enabled observations to be carried out to triangulate the data that respondents shared with the research team and enabled women to be interviewed multiple times over the course of their pregnancy to develop rapport, cross-check previous responses and to monitor their experiences of care over the course of a pregnancy with a follow-up post-delivery. However, the findings, with regard to malaria interventions, are limited by several factors. Regarding ITN use, reported use could not be confirmed with observational data at all the sites: as a result of the organization of dwellings and the restrictions on researchers’ access to sleeping spaces, only in Kenya was it regularly possible to observe the presence of ITNs in women’s sleeping quarters. Also, by relying on women’s reported malaria treatment practices – because it was not feasible to carry out direct observations of women’s drug intake outside of the health facility – malaria self-treatment and non-compliance with anti-malarial treatment courses may have been underestimated across all the sites. The lack of systematic observations of drug intake makes the cases of self-treatment and non-adherence all the more striking and highlights a need for further systematic analysis.

## Conclusions

Respondents generally valued ITNs as malaria prevention, however, availability and cost were barriers to ITNs ownership. Sleeping arrangements, climatic conditions and prioritizing infants led to inconsistent ITN use during pregnancy. Health messages could address certain issues, for example, if ITNs are left unused during pregnancy so that they are new items to mark the birth of a child.

In contrast, awareness of IPTp varied notably across the sites and, together with past experience of side effects, low awareness contributed, to non-adherence. Although IPTp was not always delivered under DOT, adherence was common and this was linked to women’s overall attempts to follow the instructions of health staff and their positive evaluation of ANC as a package of interventions.

Malaria diagnostic tests were not always available, but confirming a malaria diagnosis through a blood test was generally valued. However, there were examples of overtreatment in Ghana and Malawi: in Ghana, this resulted from misinterpretation of national policy, which illustrates the need for monitoring of local implementation. Instance of self-treatment and non-compliance with prescribed treatment courses were linked to the broad local illnesses that overlapped with malaria, the cost and availability of anti-malarials at health facilities and individual preferences – based on past and current experience of the drugs. Health messages should therefore also address adherence to prescribed anti-malarials as well as discouraging self-treatment.

## Competing interests

The authors declare that they have no competing interests.

## Authors’ contributions

CP: contributed to the overall study design; supervised and assisted with data collection in Kenya; analysed the data from this site; prepared the first draft of the manuscript and contributed to its revision based on comments from co-authors. AM: contributed to the overall study design; supervised and assisted with data collection in Ghana and Malawi; analysed the data from these sites;. provided comments on the first draft of the manuscript and contributed to its revision based on the comments from co-authors. NAA: collected data in central Ghana; revised the manuscript and provided comments. LM: collected data at the Malawi site; revised the manuscript and provided comments. SC: collected and supervised data collection in northern Ghana; revised the manuscript and provided comments. FW: collected data in Kenya; revised the manuscript and provided comments. AH: supervised data collection in northern Ghana; revised the manuscript and provided comments. MJH: supervised data collection in Kenya; revised the manuscript and provided comments. LK: supervised data collection in Malawi; revised the manuscript and provided comments. HT: supervised data collection in central Ghana; revised the manuscript and provided comments. RP: conceived and designed study; obtained project funding; provided comments and contributed to the revision of the manuscript based on comments from all co-authors. All authors: read and approved the final version of the manuscript.
